# Skin Grafting

**Published:** 1918-08

**Authors:** 


**Skin Grafting.** By J. C. MASSON, Rochester, Minn. *Journal of the American Medical Association,* vol. LXX, June 1, 1918, p. 158.

Masson emphasizes the fact that many wounds primarily infected are under modern methods made rapidly sterile and may then be closed like any fresh wound. But where there has been much skin destruction, ordinary plastic methods will not suffice, and skin grafting must be resorted to. While skin grafting was practised in ancient times it was not until 1869 following the reading of a paper by Reverdin in Paris that it was accepted as a regular surgical procedure.

Masson refers to the three types of grafts : (1) Reverdin consisting of small islets of skin, mostly epidermis but containing some dermis in the center; (2) Thiersch, consisting of sheets of skin as thin as possible, and consisting chiefly of epidermis; (3) Wolfe graft, consisting of entire thickness of skin (the most satisfactory when successfully used). There are three sources from which a graft may be obtained : (1) the patient — autoplastic grafts; (2) another person — isoplastic grafts; (3) from one of the lower animals — zooplastic grafts. Autografts are best, but Masson believes that isografts have a larger field of usefulness than is generally believed. He has used them with almost as much success as autografts, but insists that the blood of donor and recipient should be tested for agglutination, and that a donor for skin grafting should be selected in the same manner as a donor for blood transfusion; which means that besides the agglutination test, a Wasserman test should also be done.

For satisfactory skin grafting a wound should be as nearly sterile as possible, and granulation should be healthy. If not, means should be taken to improve their condition, either by curetting or excision of ulcer, or the use of various solutions, such as Dichloramin-T, or Dakin's solution.

As to the kind of graft to be used, the Wolf graft is the best, but is not so likely to take as the others. However, where possible, especially around joints, or where dealing with extensive areas, recourse should be had at least in part to grafts of the entire thickness of skin.

As to the part of the body from which to take the graft, the type of skin desired should be considered. Ordinarily the outer and anterior surface of thigh or upper arm will prove most satisfactory.

General or local anesthesia may be used. Masson finds the iodin method of skin preparation as satisfactory as any. He uses 1 : 1000 iodin in benzin, and after this dries, applies two coats of a 3.5 per cent iodin in alcohol. His method of obtaining grafts varies somewhat from the usual. He first obtains a Thiersch graft by the usual method; then, if the skin is thick, a second layer may be removed from the same area in the same way, or small island grafts may be taken from the center of the raw surface to include some of the deeper layers of epidermis and some of the superficial layers of dermis. He then reduces the size of the wound from which the grafts have been taken by excising an elliptical area of subcutaneous tissue and bringing the edges together with sutures This removed tissue may also be used if necessary by cutting it into small sectional grafts (a term used by Colebrook and Fleming) and applying it just as the ordinary Reverdin grafts are applied. By this method twice the amount of grafting material is obtained from a given area, and the suture wound heals by primary union which is an advantage over the raw area left after the removal of Thiersch and Reverdin grafts. If the wound is completely covered with Thiersch grafts, the open method is best, with the occasional removal of crusts and the application by atomizer of Dichloramin-T (4 per cent) or Dakin's solution. If the wound is only partially covered by grafts, he uses paraffin gauze over which is applied a wet dressing changed every four hours for three days. For the next three or four days he uses wet dressings at night and open air treatment by day. As a rule grafts have taken in a week's time.

